# Multi-Addressed Fiber Bragg Structures for Microwave-Photonic Sensor Systems

**DOI:** 10.3390/s20092693

**Published:** 2020-05-09

**Authors:** Oleg Morozov, Airat Sakhabutdinov, Vladimir Anfinogentov, Rinat Misbakhov, Artem Kuznetsov, Timur Agliullin

**Affiliations:** 1 Department of Radiophotonics and Microwave Technologies, Kazan National Research Technical University named after A.N. Tupolev-KAI, 10, Karl Marx st., 420111 Kazan, Tatarstan, Russia; microoil@mail.ru (O.M.); aakuznetsov@kai.ru (A.K.); taagliullin@mail.ru (T.A.); 2 Department of Special Mathematics, Kazan National Research Technical University named after A.N. Tupolev-KAI, 10, Karl Marx st., 420111 Kazan, Tatarstan, Russia; v.anfinogentov@yandex.ru; 3 Engineering Center “Computer Modeling and Engineering in the Field of Energy and Power Engineering”, Kazan State Power Engineering University, 51, Krasnoselskaya st., 420066 Kazan, Tatarstan, Russia; energy@zerdex.pro

**Keywords:** microwave-photonic sensor systems, Fiber Bragg Gratings, Addressed Fiber Bragg Structures, Multi-Addressed Fiber Bragg Structures

## Abstract

The new theory and technique of Multi-Addressed Fiber Bragg Structure (MAFBS) usage in Microwave Photonics Sensor Systems (MPSS) is presented. This theory is the logical evolution of the theory of Addressed Fiber Bragg Structure (AFBS) usage as sensors in MPSS. The mathematical model of additive response from a single MAFBS is presented. The MAFBS is a special type of Fiber Bragg Gratings (FBG), the reflection spectrum of which has three (or more) narrow notches. The frequencies of narrow notches are located in the infrared range of electromagnetic spectrum, while differences between them are located in the microwave frequency range. All cross-differences between optical frequencies of single MAFBS are called the address frequencies set. When the additive optical response from a single MAFBS, passed through an optic filter with an oblique amplitude–frequency characteristic, is received on a photodetector, the complex electrical signal, which consists of all cross-frequency beatings of all optical frequencies, which are included in this optical signal, is taken at its output. This complex electrical signal at the photodetector’s output contains enough information to determine the central frequency shift of the MAFBS. The method of address frequencies analysis with the microwave-photonic measuring conversion method, which allows us to define the central frequency shift of a single MAFBS, is discussed in the work.

## 1. Introduction

Common problems of Fiber Bragg Gratings (FBG) array interrogation in optical sensor systems are their complexity and the high cost of interrogators due to the technique of interrogation and FBG multiplexing [1,2,3,4,5]. Wavelength [1], time [2], frequency [3], polarizing [4], and spatial [5] division multiplexing requires using complex devices, such as spectrum analyzers, spectrometers with tunable Fabry–Perot interferometers, diffraction gratings, etc. All of them use the technique of optic signal receiving on charge-coupled devices with its further complex analysis. The complexity is also described by the fact that these sensors are not addressable per se, and therefore, any spectrum overlapping leads to interrogation errors [6,7,8].

In parallel with multiplexing methods and microwave photonics methods, the optical pulse-coding, phase-coding, low coherent interferometer with the cascaded FBGs methods is developed. The coding methods allow us to recognize two or more FBGs with the same spectral range [9,10,11,12]. Spectral-coding sensors are based on code-multiplexing technology [11,12], where the interrogation is produced in real time according to autocorrelation between sensor spectra and its code signature. In the series of works, it was demonstrated that the spectral-amplitude-coding method with super-structured FBG sensors, based on discrete prolate spheroidal sequences, can be useful even in the case of overlap of their optical ranges [13,14,15,16].

An easier solution was found in the Addressed Fiber Bragg Structure (AFBS) usage with the microwave photonics interrogation method [17]. The AFBS is a special type of FBG, the reflection spectrum of which has two narrow notches. The light, passed through AFBS, has two narrow optical frequencies, the difference between which is much less than an optical frequency (THz) and located in microwave range (GHz). The differential frequency is called the address frequency of an AFBS. The address frequency is invariant to stress or temperature fields; moreover, it is invariant to AFBS central frequency shifting. The AFBS in sensor systems is used both as a two-frequency source (due to the fact that it has two narrow optical frequencies with the difference between them being in the microwave range) and as a sensor of measurement system (due to the fact that its address frequency is invariant to measurable fields) simultaneously. It allows us to design a microwave-photonic sensor system based on arrays of AFBSs, on condition that the set of address frequencies in the array is orthogonal [17,18].

The evolution from AFBS to Multi-Addressed Fiber Bragg Structures (MAFBS) is logical and self-consistent. In spectral response of MAFBS, three (or more) frequency carriers are configured, and their beatings on a photodetector form three (or more) address frequencies. The combining of address frequencies allows us to expand the sensor capacity of the measurement system; moreover, it allows us to increase the accuracy of central wavelength determination.

## 2. Multi-Addressed Fiber Bragg Structure

The MAFBS (as well as AFBS) is a special type of FBG, the reflection spectrum of which has three (or more) narrow notches. The light, passed through MAFBS, has three (or more) narrow optical frequencies, the difference between which is much less than an optical frequency (THz) and is located in the microwave range (GHz). The set of all differential frequencies is named the address frequencies set of a MAFBS. The address frequencies set is invariant to strain or temperature fields, and it is also invariant to central frequency shifting [17,18,19,20]. The MAFBS is both a multi-frequency source and a sensor of the measurement system at the same time. It is necessary to require the additional conditions to use a MAFBS as a sensor in the microwave sensor system, namely: The light only from MAFBS’s narrow-band frequencies must trap into the light analysis area for the whole MAFBS central frequency shift range, which corresponds to the measurement range [17,18,19,20].

**Figure 1:**
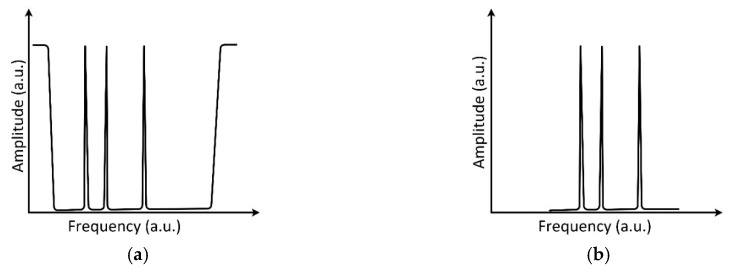
Amplitude–frequency diagrams: (**a**) transmitted through Multi-Addressed Fiber Bragg Structure (MAFBS), formed using Fiber Bragg Gratings (FBG) with π-shifts; (**b**) reflected from MAFBS, formed as a set of ultra-narrowband FBGs.

There are at least two approaches to MAFBS (as well as AFBS) forming: The first of them is the introduction of phase π-shifts into the classic FBG periodic structure [21] (Figure 1a), and the second one is the MAFBS forming as a set of ultra-narrowband FBGs [17], (Figure 1b).

## 3. Mathematical Model

**Figure 2:**
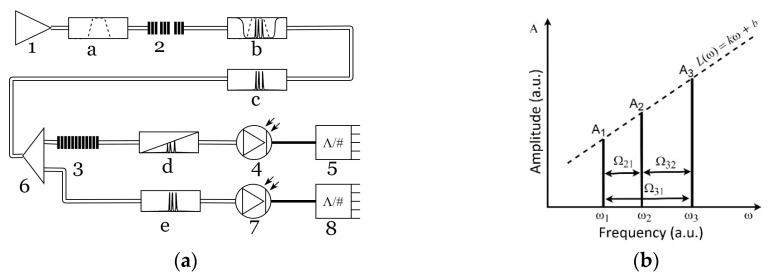
Multi-Addressed Fiber Bragg Structure: (**a**) a microwave-photonic interrogation scheme; (**b**) a spectral response of Multi-Addressed Fiber Bragg Structure.

The optoelectronic schemata, designed for light reflection and propagation, differ from each other a little, but both of them are based on the common idea. The complex light response from MAFBS, passed through optic filter with an oblique amplitude–frequency characteristic, is received on a photodetector with the subsequent analysis of output electric signal on the address frequencies set. An example of the optoelectronic scheme for light propagation is presented in Figure 2a. The optic source (1) forms a finite band light (a), which passes through MAFBS (2) and forms a multi-frequency (in this case three-frequency) signal (c); the three-frequency optic signal, passing through the optic filter with an oblique amplitude–frequency characteristic, forms an asymmetric three-frequency optic signal (d), which is received on a photodetector (4); after the photodetector, signal is received on an analog-to-digital converter (5), its subsequent analysis is performed. The system also includes the reference channel, in which the optic signal is received on a photodetector (7) directly after the MAFBS without its asymmetrical deformation in the filter (3), and then the signal is processed using the analog-to-digital converter (8). All subsequent calculations are produced with the ratios of values in the measuring and reference channels. This allows us to avoid influence of light power fluctuation, which is not connected with the MAFBS central frequency shifting.

The shape of a MAFBS spectral response at the output end of the optic filter with an oblique amplitude–frequency characteristic is shown in Figure 2b, where the following notation is used: ω_1_, ω_2_, and ω_3_ are the frequencies of optical carriers; Ω_21_ and Ω_32_ are the address frequencies; *k* and *b* are the predefined parameters of the optic filter with an oblique amplitude–frequency characteristic.

The central frequency shift of the MAFBS leads to a change of the mutual relation of the optical carriers’ amplitudes, which causes a change of the beating parameters at the address frequencies Ω_21_, Ω_32_, and their sum Ω_31_ = Ω_21_ + Ω_32_. The task is to determine the MAFBS central frequency (any of the frequencies ω_1_, ω_2_, or ω_3_), using the known parameters of the beating at the address frequencies set.

The light response from the MAFBS, which passes through the filter with an oblique amplitude–frequency response, can be written as:

(1) $$S(t)=A_{1}\sin(\omega_{1}t+\varphi_{1})+A_{2}\sin(\omega_{2}t+\varphi_{2})+A_{3}\sin(\omega_{3}t+\varphi_{3}),$$

where *A*_1_, *A*_2_, and *A*_3_ are the amplitudes, φ_1_, φ_2_, and φ_3_ are the initial phases of the signal at the optical frequencies ω_1_, ω_2_, and ω_3_. The output current of the photodetector *F*(*t*) is proportional to the square of the optic response:

(2) $$\begin{array}{l} F(t)\sim A_{1}^{2}+A_{2}^{2}+A_{3}^{2}+2A_{1}A_{2}\cos(\Omega_{21}t+\varphi_{2}-\varphi_{1})+\\ \;+2A_{2}A_{3}\cos(\Omega_{32}t+\varphi_{3}-\varphi_{2})+2A_{1}A_{3}\cos(\Omega_{31}t+\varphi_{3}-\varphi_{1}), \end{array}$$

in which the oscillations at optical (terahertz) frequencies are excluded [17,18]. The known values in (2) are the address frequencies Ω_21_ and Ω_32_. The constant signal level in (2), the amplitudes at the address frequencies Ω_21_, Ω_32_, and their sum Ω_31_ give four independent equations for the determination of three unknown amplitudes, *A*_1_, *A*_2_, and *A*_3_:

(3) $$\left\{ \begin{array}{l} D_{0}=A_{1}^{2}+A_{2}^{2}+A_{3}^{2}\\ D_{21}=2A_{1}A_{2}\\ D_{32}=2A_{2}A_{3}\\ D_{31}=2A_{1}A_{3} \end{array}\right.,$$

where *D*_0_, *D*_21_, *D*_32_, and *D*_31_ are the measured values of constant signal level, amplitudes of the address frequencies Ω_21_, Ω_32_, and their sum Ω_31_, respectively.

The resulting system of four equations is overdetermined, since the number of equations exceeds the number of unknowns. Moreover, Equation (3) must be supplemented by the requirements that the points (ω_1_, *A*_1_), (ω_2_, *A*_2_), and (ω_3_, *A*_3_) belong to the same line:

(4) L(ω)=k·ω+b,

which describes the filter with an oblique amplitude–frequency characteristic. Moreover, it is necessary to require that the differences ω_2_ − ω_1_ and ω_3_ − ω_2_ are equal to the address frequencies Ω_21_ and Ω_32_, respectively, and the condition ω_3_ − ω_1_ = Ω_32_ + Ω_21_ would also be automatically satisfied. Thus, it is necessary to add the additional relation to Equation (3):

(5) $$\frac{A_{2}-A_{1}}{\Omega_{21}}=\frac{A_{3}-A_{2}}{\Omega_{32}},$$

which binds the task parameters, imposing restrictions on finding a solution, while simultaneously describing the mutual relations between the frequencies. Having the amplitudes *A*_1_, *A*_2_, and *A*_3_ from Equation (3), supplemented by Equation (5), and using the known values of the parameters *k* and *b* of the filter with an oblique linear amplitude–frequency characteristic, one can calculate the MAFBS frequencies ω_1_, ω_2_, and ω_3_.

There are different ways to define the MAFBS central frequency, since MAFBS has three narrow resonances in addition to its main Bragg resonance. We used the definition of the central frequency of MAFBS as an average, according to the formula:

(6) $$\omega_{Br}=\frac{1}{2}\left(\frac{\omega_{1}+\omega_{2}}{2}+\frac{\omega_{2}+\omega_{3}}{2}\right)=\frac{1}{4}(\omega_{1}+2\omega_{2}+\omega_{3}),$$

which uniquely determines the position of the MAFBS.

The overdetermined equations system can be solved by searching the conditional extremum of the function:

(7) $$\Phi (A_{1},A_{2},A_{3},\lambda )={\left(A_{1}^{2}+A_{2}^{2}+A_{3}^{2}-D_{0}\right)}^{2}+{\left(D_{12}-2A_{1}A_{2}\right)}^{2}+{\left(D_{23}-2A_{2}A_{3}\right)}^{2}+{\left(D_{13}+2A_{1}A_{3}\right)}^{2},$$

relatively to the restriction:

(8) $$f(A_{1},A_{2},A_{3})=(A_{2}-A_{1})\Omega_{32}-(A_{3}-A_{2})\Omega_{21}=0,$$

requiring a minimum of the Lagrange function, expressed in the form:

(9) $$\Psi (A_{1},A_{2},A_{3},\lambda )=\Phi (A_{1},A_{2},A_{3})+\lambda \cdot f(A_{1},A_{2},A_{3})\to \min,$$

where λ is the Lagrange multiplier.

Equation (9) is equivalent to the requirement that all partial derivatives with respect to the variables *A*_1_, *A*_2_, *A*_3_, and λ are equal to zero, which leads to a set of four non-linear equations:

(10) $$\left\{ \begin{array}{l} \partial \Psi /\partial A_{1}=\partial \Phi /\partial A_{1}-\lambda \Omega_{32}=0\\ \partial \Psi /\partial A_{2}=\partial \Phi /\partial A_{2}+\lambda (\Omega_{21}+\Omega_{32})=0\\ \partial \Psi /\partial A_{3}=\partial \Phi /\partial A_{3}-\lambda \Omega_{21}=0\\ \partial \Psi /\partial \lambda =(A_{2}-A_{1})\Omega_{32}-(A_{3}-A_{2})\Omega_{21} \end{array}\right.,$$

where the partial derivatives ∂Φ/∂*A_i_* are not expressed due to their obvious simplicity. 

Non-linear Equation (10) (due to the nonlinearity of the partial derivatives ∂Φ/∂*A_i_*, *i* = 1, 2, 3) can be solved only numerically. The values *A*_1_, *A*_2_, and *A*_3_, which are the solution of Equation (3) with the exception of the first equation, can be taken as the initial conditions, and λ can be taken as the initial value equal to zero:

(11) $$A_{01}=\sqrt{\frac{D_{31}D_{21}}{2D_{32}}},\;A_{02}=\sqrt{\frac{D_{21}D_{32}}{2D_{31}}},\;A_{03}=\sqrt{\frac{D_{31}D_{32}}{2D_{21}}},\;\lambda_{0}=0.$$

After that, Equation (10), supplemented by the initial values in Equation (11), is solved by any well-converging iterative method, for example, the Levenberg–Marquardt [22,23] or Newton–Raphson [24] methods.

Equation (10) solution gives the values of the amplitudes *A*_1_, *A*_2_, and *A*_3_, each of which can be used to determine the MAFBS central frequency position relative to the filter with an oblique amplitude–frequency characteristic. Substituting the found values of the amplitudes *A*_1_, *A*_2_, and *A*_3_ in Equation (4), and combining them in Equation (6), we obtain the expression for the central frequency of the MAFBS:

(12) $$\omega_{Br}(D_{0},D_{12},D_{13},D_{32})=\frac{1}{4k}\left(A_{1}+A_{2}+A_{3}-3b\right),$$

as a function of the measured values of *D*_0_, *D*_21_, *D*_32_, and *D*_31_—a constant signal level, and amplitudes at address frequencies Ω_21_, Ω_32_, and their sum Ω_31_, respectively.

## 4. Generalized Modulation Factor

An alternative solution for MAFBS central frequency determination can be obtained through a generalization of the modulation factor of the output current after the photodetector (Equation (2)), which for a two-address MAFBS can be written as follows:

(13) $$M(\omega )=\frac{A_{1}(\omega )A_{2}(\omega )+A_{1}(\omega )A_{3}(\omega )+A_{2}(\omega )A_{3}(\omega )}{A_{1}^{2}(\omega )+A_{2}^{2}(\omega )+A_{3}^{2}(\omega )}.$$

Note that in the particular case, when one of the amplitudes is equal to zero, the modulation factor coincides with the modulation factor of two-frequency beatings within a constant factor [18,19,20]. Equation (13) is a monotonic function of the MAFBS central frequency shifting relative to a filter with an oblique amplitude–frequency characteristic. The monotonicity of the generalized modulation factor in Equation (13) allows us to use it as single measured parameter for central frequency determination.

## 5. Results of Numerical Modeling

There is no detection limit of the central wavelength shift of the MAFBS in the numerical model, but it depends on an analog-to-digital converter accuracy. We use the classical approach to transform equations system into dimensionless quantities, and the characteristic quantities are defined as dimensional frequency Ω^0^ and dimensional amplitude *A*^0^. As the characteristic dimensional frequency of task Ω^0^, we set the frequency corresponding to 125 GHz (in wavelength terms, it is 1 nm). The characteristic dimensional amplitude *A*^0^ depends on the maximum output current of the photodetector, which can be independently amplified or attenuated to any value. We normalize all the task variables, so that the maximum central frequency shifting of the MAFBS does not exceed 125 GHz or Ω^0^, which is equal to 1 conventional unit. We normalize the maximum signal amplitude, so that in dimensionless quantities the maximum signal level does not exceed 1000 conventional units. Based on this, we define the parameters of optic filter with an oblique linear amplitude–frequency characteristic as *k* = 1000 conventional units and *b* = 100 conventional units. As the MAFBS model, we choose a structure with address frequencies Ω_21_ = 0.01 conventional units (1.25 GHz) and Ω_32_ = 0.02 conventional units (2.50 GHz), with a range of changes in the MAFBS central frequency to 1 conventional unit.

The relative error in determining the MAFBS central frequency is determined by the formula:

(14) $$\varepsilon (D_{0},D_{12},D_{13},D_{23},E_{F})=\frac{\left|{\hat{\omega }}_{Br}-\omega_{Br}\right|}{\omega_{Br}}.$$

where ${\hat{\omega }}_{Br}={\hat{\omega }}_{Br}(D_{0},D_{12},D_{13},D_{32})$ is the central frequency of the MAFBS, calculated without error, and $\omega_{Br}=\omega_{Br}(D_{0},D_{12},D_{13},D_{32},E_{F})$ is the central frequency, calculated with the error *E*_F_ of determining the amplitudes *D*_0_, *D*_21_, *D*_32_ and *D*_31_.

**Figure 3:**
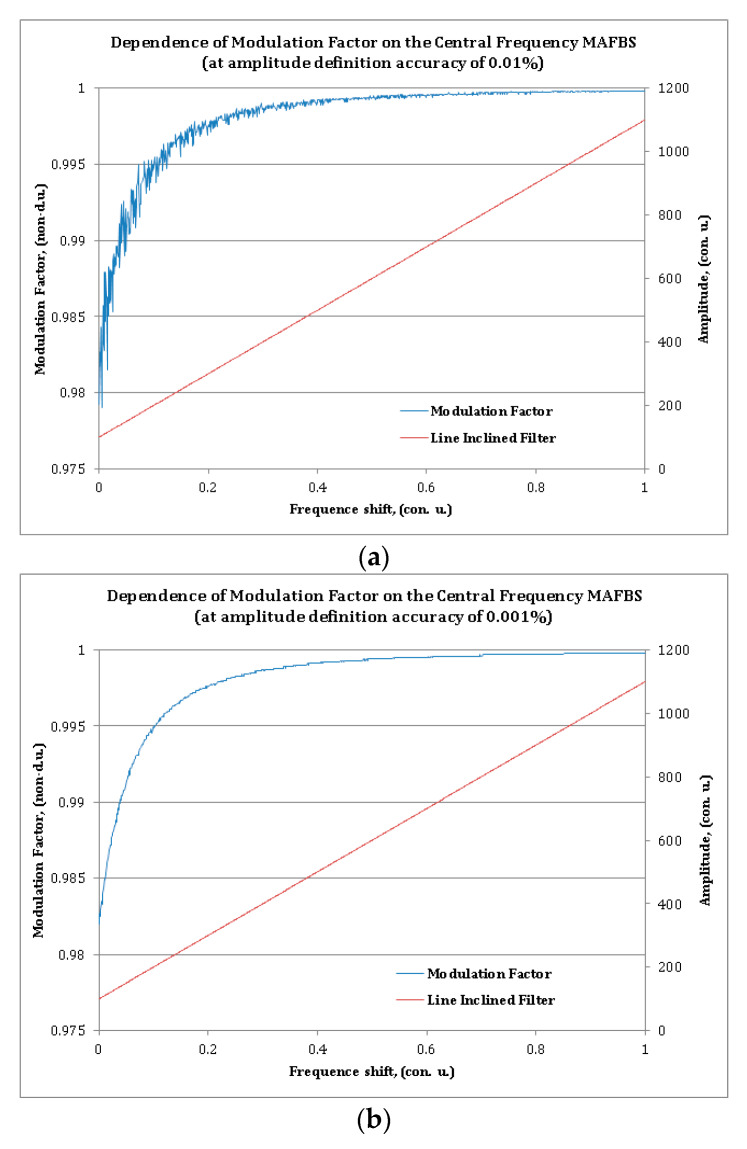
Modulation factor as the function of MAFBS central frequency shifting: The blue line is the dependence of the modulation factor; the red line is the spectral characteristic of the optic linear oblique filter, for an amplitude definition accuracy of (**a**) 0.01% and (**b**) 0.001%.

All simulations were made for two values of an amplitude determination error—with the error *E*_F_ of 0.01% and 0.001% of the full scale. The dependence of the modulation factor on the central frequency shift of the MAFBS at amplitude determination error equal to 0.01% is shown in Figure 3a, and at 0.001% is shown in Figure 3b. The blue line indicates the modulation factor, and the red line is the spectral response of the filter with an oblique linear amplitude–frequency characteristic. As can be seen from the Figure 3, only high-precision amplitude measurement of the photodetector output current leads to acceptable accuracy of the MAFBS central frequency shift determination. Due to this fact, the subsequent development of microwave-photonic measuring systems based on the generalized modulation factor is a very difficult task, since it requires high accuracy in the amplitude determination of the output current of the photodetector. The relative error of MAFBS central frequency shifting determination does not exceed 10^−1^ and 10^−2^ for *E_F_* = 0.01% and 0.01%, respectively. These values cannot be considered acceptable for high-precision measurements.

The second conclusion that follows from the modulation factor dependence is that this dependence does not provide uniformity of the measurement scale in the whole measurement range. The task of regularizing the measurement scale can be solved [17]; however, it requires additional complication of the optoelectronic scheme.

**Figure 4:**
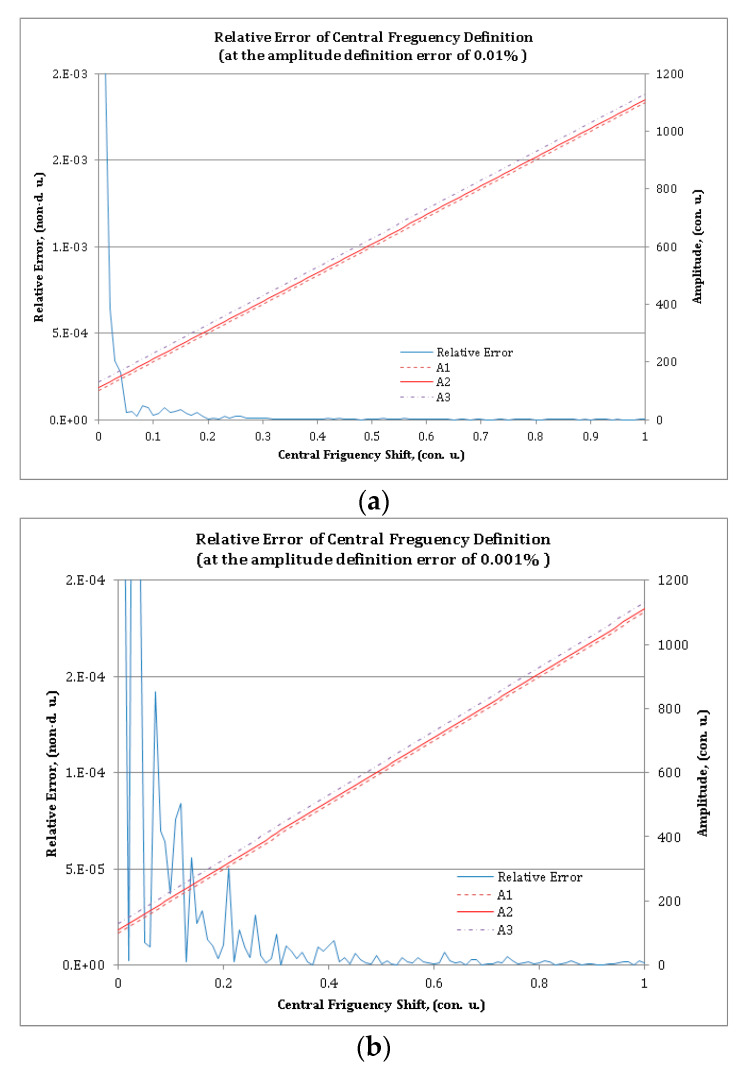
Relative error of the MAFBS central frequency definition for the amplitude definition error of (**a**) 0.01% and (**b**) 0.001%: The thick line is the relative error; the thin lines are the calculated amplitudes of the frequency components.

In Figure 4, violet, red, and brown lines show the calculated amplitudes of the carrier frequencies of the MAFBS obtained by numerically solving Equation (10) with the initial values in Equation (11). The blue line shows the relative error of central frequency shift determination of the MAFBS. Two calculation sets for *E*_F_ = 0.01% (Figure 4a) and *E*_F_ = 0.001% (Figure 4b) were made. The relative error of the central frequency shift determination of MAFBS, calculated via Equation (13), does not exceed 10^−3^ (for *E*_F_ = 0.01%) and 10^−4^ (for *E*_F_ = 0.001%), almost in the whole measurement range. The only exception is a small sector, where the amplitudes (*A*_1_, *A*_2_, and *A*_3_) are close to zero.

The simulation results clearly demonstrate that the proposed method of the MAFBS central frequency determination is two orders of magnitude more precise than the method based on the generalized modulation factor.

## 6. Conclusions

Studies have confirmed the perspectivity of MAFBS usage instead of AFBS for Microwave Photonics Sensor Systems (MPSS). The MAFBS multi-addressing idea is that in FBG structure, three (or more) narrow optical frequencies are formed, the difference between which is much less than an optical frequency (THz) and is located in the microwave range (GHz). The task of strict definition of central frequency shifting of MAFBS with the given address frequencies set is specified and solved. The equations system, which describes photodetector output current dependence from MAFBS central frequency shifting relative to the optic filter with oblique characteristics parameters, is written. The additional restrictions, connected with a microwave-photonic interrogation method and an optoelectronic scheme, were added to this equations system. We suggest the mathematical model based on this equations system, and the method of its solution, which allows us to define the central frequency shift unequivocally, according to the output current of a photodetector in MPSS, and as a result to define the values of applied physical fields. We made an attempt to use the generalization of modulation factor as a single measuring parameter to correlate it with the MAFBS central frequency shifting. It was found that generalization of modulation factor usage has a lot of disadvantages, since it requires high measurement accuracy of photodetector output current parameters, thus making this method unattractive.

Based on the mathematical model and its simulation, the task of strict definition of central frequency shifting of MAFBS, when the parameters of output photodetector current are measured with errors, was solved. It was shown that the suggested method of MAFBS usage in MPSS satisfies accuracy requirements, has potential for development, and allows us to move to an experimental research step.
